# Strategical Ways for Dental Anxiety Management Prior to Third Molar Extraction

**DOI:** 10.7759/cureus.68023

**Published:** 2024-08-28

**Authors:** Turki A Alshehri, Shahad T Alameer, Abdullah M Almotreb

**Affiliations:** 1 College of Dentistry, Imam Abdulrahman Bin Faisal University, Dammam, SAU; 2 Dentistry, Riyadh Specialized Dental Center, Riyadh Second Health Cluster, Riyadh, SAU

**Keywords:** anxiety management, dental management, third molar, dental extraction, dental anxiety

## Abstract

A patient experiencing moderate to severe anxiety often skips regular check-ups, choosing to visit only when necessary. As a result, their oral health worsens. Various variables can affect anxiety such as age, gender, culture, and previous experiences. One of the most frequent oral surgical procedures is extracting the third molar, which is accompanied by severe pain, tension, and anxiety, the use of invasive equipment, and local anesthetic. A combined effect was found of four factors, including the patient, dental employees, setting, and dental procedure, which can trigger and affect dental anxiety. Both pharmacological and non-pharmacological approaches were explored; as a non-drug approach, anxiety was managed by music therapy. Acupuncture, music, audio-visual aids, and lavender oil showed an effect that helped in reducing anxiety. Patients’ dental anxiety can be reduced through behavioral management such as educating the patients before the surgical procedure, relaxation strategies, biofeedback, and exposure therapy. Medications include benzodiazepines, nitrous oxide (N_2_O), and midazolam. Effective treatment relies on collaboration between the patients and dentists; calm patients not only provide a manageable environment for the dental team but can also result in better treatment outcomes.

## Introduction and background

Tooth extraction is among the most common procedures carried out in oral surgery. The primary reasons for extraction typically include advanced decay, pericoronitis, formation of cysts, periodontal issues, and removal needed for orthodontic treatments [1]. A patient experiencing moderate to intense anxiety is inclined to skip regular check-ups, choosing to visit only when necessary. This corresponds to the patient’s oral health often getting worse and visiting the clinic only when the condition is advanced [2,3]. It has been approximated that between 6% and 14% of Americans postpone getting dental care out of fear [4]. It was also estimated that one in seven patients receiving treatment in Western nations has significant levels of fear or anxiety [3]. Age, gender, culture, and previous experiences are variables that affect anxiety [5].

One of the most common oral surgical procedures is the extraction of the third molar, which often goes along with significant pain, stress, and anxiety, as well as the use of invasive tools and local anesthesia [6]. It has been determined that preoperative anxiety is linked to greater postoperative pain and longer surgical treatment [7]. Anxiety can cause an increase in heart rate and blood pressure, pallor, profuse perspiration, dizziness, and a fight-or-flight response as a physiological reaction to stressful situations [8]. The autonomic nervous system’s activity varies because of dental anxiety. For example, alterations in the autonomic nervous system's function during dental procedures can lead to medical emergencies such as elevated blood pressure and vasovagal responses. For the safe execution of dental treatments, dentists must comprehend the degree of a patient’s preoperative anxiety and the activity level of their autonomic nervous system [9]. It was found that the combined effect of four factors - the patient, dental employees, the setting, and the dental procedure - can both trigger and affect dental anxiety [9].

The ‘4 Ss’ are four main factors that can cause dental anxiety: sights (such as drills and injections), sounds (such as drilling and extraction breaking sounds), sensations (such as vibrations), and scents (such as eugenol and hypochlorite) [10]. To make their recovery as comfortable and quick as possible, it is crucial to aid patients in managing their preoperative anxiety and enduring the postoperative phase with the least amount of discomfort [11]. Non-pharmacological management for anxiety has been managed with music therapy as a non-drug approach [12]. Patients’ dental anxiety can be reduced through behavioral techniques, such as preoperative education, relaxation techniques, biofeedback coaching, and exposure treatment, as explained among the age group between 18-40 years [11]. In terms of pharmacological approaches, several medications, including benzodiazepines, nitrous oxide (N2O), opioids, barbiturates, alpha-2 adrenergic receptor agonists, phytotherapies, and others, have been suggested for the management of dental anxiety [8]. However, the use of pharmacological treatments, such as sedation or general anesthesia, carries some significant consequences and is often contraindicated for individuals who also have specific medical conditions. Medical professionals as well as patients are inclined to use alternative means of reducing stress and reducing anxiety [11].

The most common approach for significantly decreasing anxiety both before and during surgery is to provide preoperative information outlining the specifics of the surgical operation and the anticipated postoperative recovery period [11]. To obtain the patient’s informed consent, the patient must be given enough information. According to the General Medical Council of the UK, this information should include an understanding of the diagnosis, prognosis, and treatment options, as well as specifics regarding the treatment process, its objective, anticipated advantages, frequent side effects, and potentially serious complications that may arise. Additionally, it has been noted that the third most frequent anxiety-inducing element in the lead-up to oral surgery was providing the patient with insufficient information about the treatment process [13]. It was also reported that patients who had undergone oral surgery in the past reportedly experienced less anxiety than those who had never had the procedure [14]. It is crucial to manage anxious patients effectively when they seek dental treatment. The dentist should work to minimize patients’ fear and anxiety to encourage those patients to attend dental appointments in the future. This review provides an overview of dental anxiety and possible treatment options for adults.

## Review

There are multiple approaches to reduce anxiety before tooth extraction. 

Music

Music is frequently used to reduce tension and anxiety during dental procedures [15]. Due to its suppressive influence on the sympathetic nervous system, which results in a decrease in both adrenergic activity and neuromuscular activation, music therapy has been utilized as a non-pharmacological technique to regulate anxiety [12]. A clinical study concluded that while listening to music and having the mandibular third molar extracted, sympathetic nerve activity was reduced throughout incision and flap reflection, bone removal, crown separation, and extraction [15]. Another study investigated the relationship between music frequency and anxiety level during extraction. It was concluded that without affecting the patients' clinical perception of anxiety, a 432-Hz musical intervention during dental treatment was found to lower salivary cortisol levels compared to its absence. It was also shown that the clinical sense of anxiety of the participants in the music intervention groups was significantly lower than the control group. As a result, medical music therapy might be seen as a non-invasive, affordable, and efficient solution to lower patients' anxiety levels before receiving dental treatment [12]. This shows that it might be quite beneficial to the patient's anxiety levels to listen to music while the impacted third tooth is being extracted from the mandible [15].

Audiovisual aids and patient-dentist communication

The general recommendation is to connect with patients verbally, in writing, via video, or by combining any of these approaches. It has been shown that giving preoperative information to patients allows them to be less anxious during dentoalveolar surgery [2]. According to a clinical study, communicating with the patient before surgery is more successful than afterward [16]. It was shown that patients who received written information before surgery reported considerably lower postoperative anxiety levels than those who did not [2]. An assessment of patient-dentist communication via social media was carried out, and it was found that the ideal time for communication is before the procedure. This finding may be explained by the fact that answering questions aligns better with preparation than the memory reconstruction strategy [16]. In therapeutic situations, distraction techniques like virtual reality have been utilized to ease anxiety. It is a type of human-computer interaction in which the patient can experience tactile, auditory, and visual inputs through a headset to feel like part of an electronically controlled virtual environment [17]. Patients in the study reported that the virtual reality system kept them from focusing on the surgery, which helped them feel less anxious [17]. However, a small percentage of participants experienced a sense of disconnection because they were unable to observe what was surrounding them [17]. Another research assessed the preoperative education effect on reducing anxiety. It was proven that watching an educational video regarding patients' perspectives during surgery along with suggestions for relaxation significantly lowers dental anxiety in patients undergoing third molar extraction [18]. The video provided information, reassurance, and/or relaxation tips; thus, any of these elements may have contributed to the decrease in anxiety [18]. The majority of preoperative videos feature actual or simulated surgical procedures, which could be detrimental to stress management [18]. Preoperative education about third molar extraction and video relaxation techniques greatly lower patient anxiety during the procedure. Even if some patients have prior experience, all patients must receive sufficient details on the third molar extraction surgery. Giving patients thorough information can reduce postoperative anxiety [2].

Lavender oil

For decades, numerous aromatic essential oils have been utilized to treat a wide range of diseases. One of the most popular and efficient oils is lavender oil. Since ancient times, extracts from the lavender genus have been utilized in traditional medicine. Lavender oil has also been utilized as an anti-inflammatory, antiseptic, mucolytic, and carminative phytotherapeutic agent to treat minor medical conditions. One of the best essential oils for treating anxiety is lavender oil, which is recognized as a readily available and secure remedy. It has been demonstrated to treat anxiety disorders in a way that is comparable to lorazepam. Nevertheless, constant stimulation impairs cognition and eventually renders the central nervous system insensitive to all stimuli. The administration of the preferred protocol for three minutes may reduce the brain's adaption mechanism. Along with the beneficial effects, some unfavorable reactions have also been noted, particularly after repeated topical applications of specific essential oils such as increased estrogenic activity. Patients with dental anxiety who are unable to undergo general anesthesia may benefit from lavender oil, and it can lower the need for anti-psychotic medicines. Lavender oil is frequently utilized in dental offices as an alternate form of therapy or premedication because it is thought to be a relaxing agent and is preferred notably in patients with anxiety-related tension [19].

Eye coverage

Symptoms of dental anxiety can be managed by limiting exposure to any of the root causes. The Centers for Disease Control and Prevention (CDC) advises using surgical drapes to protect the surgical site from infection. Blades, drills, elevators, and other dental anxiety-inducing visual cues are eliminated while the eyes are covered by the drape. The patient can become unaware of what is happening. This lack of awareness may cause anxiety since the patient is anticipating an unknown danger, which may make them worry about the worst-case scenario. According to a clinical study, concealing the patients' eyes during surgical extraction of their third molars may worsen dental anxiety in people with moderate dental anxiety [10].

Acupuncture

To reduce side effects and prevent overusing medications, acupuncture has been employed as an alternative therapy in dentistry; this approach is both secure and efficient. At 48 and 72 hours after surgery, the mean pain levels in a clinical trial were lower in the acupuncture group than in the placebo group, and the pain was gradually reduced. However, the groups were not statistically significant. Results show that acupuncture has superior effects in reducing postoperative edema when compared to placebo acupuncture treatment, and there was no significant difference between the protocols to reduce pain, trismus, or anxiety [1].

Sedation

Sedation is one of the effective ways to reduce patients' anxiety before dental extraction. Management of dental anxiety among patients seeking third molar extraction has been illustrated by de Moares et al. who investigated different protocols that can lower dental anxiety and increase patient satisfaction at the end of the surgical procedure [4]. Three groups were distributed in this randomized clinical trial (RCT): the first group with 5 mg of diazepam orally 30 minutes before the beginning of dental extraction, the second group with 7.5 mg of midazolam orally 30 minutes before the beginning, and the third group was sedation with 40% nitrous oxide and 60% oxygen by inhalation using a Porter MXR 3000 flowmeter (Parker Hannifin, Hatfield, PA, USA) 5 minutes before dental extraction. The study concluded that all three groups showed effective results and reduced anxiety in the diazepam group followed by midazolam, whereas nitrous oxide was the lowest [4].

## Conclusions

Dental anxiety of third molar extraction can negatively affect a person's quality of life and keep them away from receiving essential dental care. Multiple pharmacological and non-pharmacological therapy techniques can be used for anxiety management of third molar extraction. Music was found to be non-invasive and reasonably priced. Before the treatment, effective interaction between the patient and the dentist has shown significantly reduced anxiety levels. Acupuncture and lavender oil have both been shown to help individuals feel less anxious; however, eye coverage during extraction must be avoided. Cooperation between the dentist and patient is one of the essential factors to keep the patient calm for better dental treatment and a relaxed environment.
